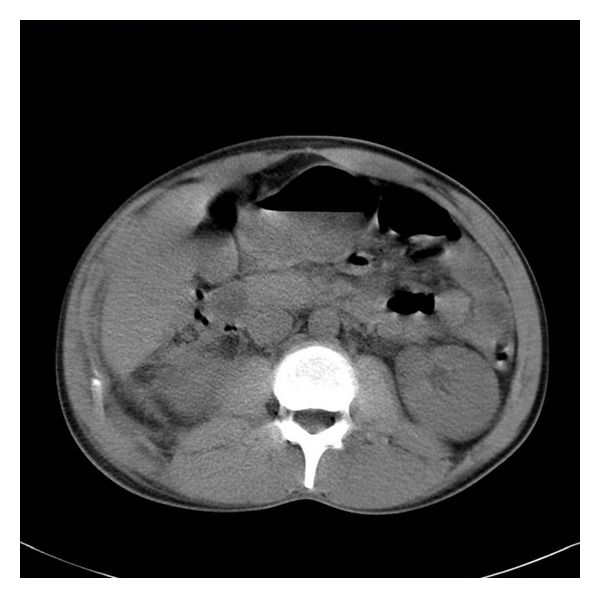# Missed Appendicitis: Mimicking Urologic Symptoms

**DOI:** 10.1155/2012/571037

**Published:** 2012-12-26

**Authors:** Hamed Akhavizadegan

**Affiliations:** Urology Department, Baharloo Hospital, Tehran University of Medical Sciences, Behdari Street, Rahahan Square, Tehran 1339973111, Iran

## Abstract

Appendicitis, a common disease, has different presentations. This has made its diagnosis difficult. This paper aims to present two cases of missed appendicitis with completely urologic presentation and the way that helped us to reach the correct diagnosis. The first case with symptoms fully related to kidney and the second mimicking epididymorchitis hindered prompt diagnosis. Right site of the pain, relapsing fever, frequent physical examination, and resistance to medical treatment were main clues which help us to make correct diagnosis.

## 1. Introduction

It is a rule in every medical ward to warn medical students about missing appendicitis, a lethal mistake. However, it is not very uncommon to see the complications of perforated appendicitis, missed by trained specialists. So writing about appendicitis is old, however, not useless. Appendicitis is great mimicker, as it was said by William Osler for Syphilis. This paper is to present two cases that can exemplify this reality. 

## 2. First Case Presentation

A twenty-five-year-old man was admitted to the emergency room with fever and severe right flank pain. After the initial evaluation and resuscitation, cultures were provided and empirical antibiotic therapy with ceftriaxone was started. He had a history of stone passing two years ago, thus in this episode, he had tried analgesics, hydration, and exercise four days before his current admission. Nevertheless, the fever had made him seek medical visit. His chief complaint was severe right flank pain which was too intense to let him pose for assessment of costovertebral angle tenderness and very little pain if any in right lower quadrant (RLQ). The ultrasound of kidney, ureter, and bladder revealed only a 3 mm calyceal stone. Spiral CT scan without contrast of abdomen and pelvis was requested to detect any probable small missed ureteral stones. The CT scan did not reveal any ureteral stones; however, right perinephric fat was unusual, losing the sharp normal lower border (Figure 1). For ruling out the diagnosis of forniced rupture, an intravenous pyelography (IVP) was ordered. In the second day of admission, the patient's body temperature returned to normal and IVP was normal, but the flank pain extremely increased. The cultures were negative and U/A was inactive. Afterwards, the patient developed fever again. Reexamination showed peritoneal irritation localized to RLQ. In laparotomy, perforated retrocecal appendicitis with draining pus to the jerota was revealed. After appendectomy, because of massive volume of pus coming out of jerota, a three-way urinary catheter was inserted into jerota and irrigation was started as in prostatectomy. For three days, pus was being drained from the indwelling catheter. The patient was discharged with good condition.

## 3. Second Case Presentation

A fourteen-year-old boy with swelling of the right testis, erythema, pain, and slight fever was admitted with the diagnosis of right epididymorchitis. The cultures were provided and antibiotics were administered. The next day fever and pain were discontinued. In ultrasound examination, some calcifications and mixed echo mass lesion in right testis were revealed. The patient had experienced a right testis trauma three months before with hematoma which was resolving gradually but his testis had not reached the normal baseline size at all. The following day, another episode of fever was started. In Reexamination, RLQ tenderness, rebound, and guarding were found. In laparotomy a pelvic appendicitis pending perforation near the pelvic vasa was excised. The erythema and pain were improved; however, the unusual large size of testis did not change. Radical orchiectomy was performed because of missed testis tumor which was confirmed by pathology. 

## 4. Discussion

In first case, retrocecal appendicitis presented with flank pain. The perforation of appendicitis did not change the presentation because pus was drained retroperitoneally over the Psoas muscle. Moreover, involvement of Jerota fascia in this process had caused more prominent flank symptoms. CT scanning with abnormal feature of right kidney confused the urologist and completely had directed him to a renal problem. If the pus had not opened its way to peritoneum and had not caused peritoneal symptoms, it would have ended in necrotizing fasciitis of Psoas muscle or prerenal abscess.

In second case, a missed testis tumor changed the patient picture. Because of obviously swelled testis, erythema, and tenderness, the pain over right lower quadrant was assumed to referred testis pain. 

In these two cases, in addition to right side of the pain, symptom resistance and relapsing fever were the main clues to reach the correct diagnosis. The key factor to avoid missing appendicitis is frequent physical examination. In spite of new diagnostic modalities [1, 2], frequent visits and abdominal examination [2] are the best way to avoid misdiagnosis. 

## Figures and Tables

**Figure 1:**